# Acquiring new complex endoscopic skills: Experience from the development of peroral endoscopic myotomy (POEM) in Malaysia

**DOI:** 10.1002/jgh3.12598

**Published:** 2021-06-25

**Authors:** Shiaw Hooi Ho, Nik M A Nik Arsyad, Peng Choong Lau, Fadhil H Jamaludin, Sanjiv Mahadeva

**Affiliations:** ^1^ Division of Gastroenterology, Department of Medicine, Faculty of Medicine University of Malaya Kuala Lumpur Malaysia; ^2^ Department of Medicine International Islamic University Malaysia Kuantan Malaysia; ^3^ Department of Surgery, Faculty of Medicine University of Malaya Kuala Lumpur Malaysia; ^4^ Department of Surgery Pantai Hospital Kuala Lumpur Kuala Lumpur Malaysia; ^5^ Department of Anaethesia, Faculty of Medicine University of Malaya Kuala Lumpur Malaysia

## Abstract

Peroral endoscopic myotomy (POEM) has rapidly gained popularity as an effective treatment modality for achalasia. However, POEM services in the South East Asian region are not widely available due to either a lack of expertise or interest. In this article, we describe how a POEM service can be developed through a combination of networking with regional experts, having prior experience of endoscopic submucosal dissection (ESD), attending animal model workshops, collaborating with upper gastrointestinal surgeons, and working together in a multidisciplinary team. A total of 68 POEM procedures have been performed since 2015, with a 94.1% technical and 93.4% clinical success rate, and a 21.5% minor complication rate. We believe that our model may be useful for other Endoscopy Units in the region, which are performing advanced therapeutic endoscopy, to develop a POEM service too.

## Graphical abstract

Increasing numbers of peroral endoscopic myotomy procedures after the development of a multidisciplinary service at the University Malaya Medical Centre.

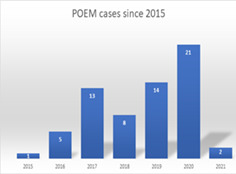

## Introduction

Esophageal achalasia is a motility disorder of unknown cause, characterized by loss of esophageal peristalsis and failure of lower esophageal sphincter (LES) relaxation in response to swallowing. Patients with achalasia typically experience dysphagia, regurgitation, retrosternal chest pain, and weight loss, and its prevalence is thought to be between 6.3 and 15.3 per 100 000 population.^1^ Although noninvasive mechanical stretching of the LES with balloon dilatation or botulinum toxin injection are recognized treatment modalities, their durability is recognized to be inferior to division/disruption of the LES mechanism—a.k.a. myotomy.^1^, ^2^ Transthoracic or laparoscopic Heller's myotomy has been the standard therapeutic approach to myotomy prior to the introduction of peroral endoscopic myotomy (POEM).

POEM was developed as an alternative to Heller's myotomy.^3^ A meta‐analysis of 15 studies (1213 patients) comparing POEM and Heller's myotomy showed that symptom improvement and the length of myotomy were greater in the former (i.e. POEM group) over a follow‐up duration of approximately 2 years.^4^ Although patients post‐POEM had more erosive esophagitis, there were neither differences in reflux symptoms nor pathologic reflux on pH monitoring between the groups. The 2019 Seoul Consensus on Esophageal Achalasia guidelines have recently recommended POEM over Heller's myotomy in type III achalasia and POEM is now the therapy of choice for recurrence of symptoms, following a failed initial pneumatic dilatation or Heller's myotomy.^1^

The first POEM was successfully performed in Japan in 2008 by Professor Haruhiro Inoue.^3^ Since then, there has been a tremendous interest and increase in the number of POEM procedures performed worldwide, with thousands of procedures reported in the literature^5^, ^6^, ^7^ Despite its popularity in East Asia^5^, ^6^ and in the United States,^7^ there has a been a dearth of POEM reports from South East Asia. We believe this may be due to a lack of expertise or possibly a lack of interest. This article details the development of a POEM service in Malaysia, and the combination of expertise that helped to initiate it.

## Development of POEM in Malaysia

In Malaysia, the POEM procedure had not been performed prior to 2013, due to a lack of expertise. The University Malaya Medical Centre in Kuala Lumpur had been organizing Live Therapeutic Gastrointestinal (GI) Endoscopy Workshops on an annual basis since 2000, under the visionary leadership of Prof Khean Lee Goh.^8^ These live endoscopy workshops would usually highlight some of the latest advancements in GI endoscopy over 2 days, performed by leading international experts. During the 2013 Live Endoscopy Workshop, two POEM procedures were performed for the first time, in two patients with achalasia by Professors Phillip Chiu (from Hong Kong) and Horst Neuhaus (from Germany) respectively.^9^ An interest in POEM had developed locally after these two procedures, but an opportunity to learn the technique was lacking.

**Figure 1:**
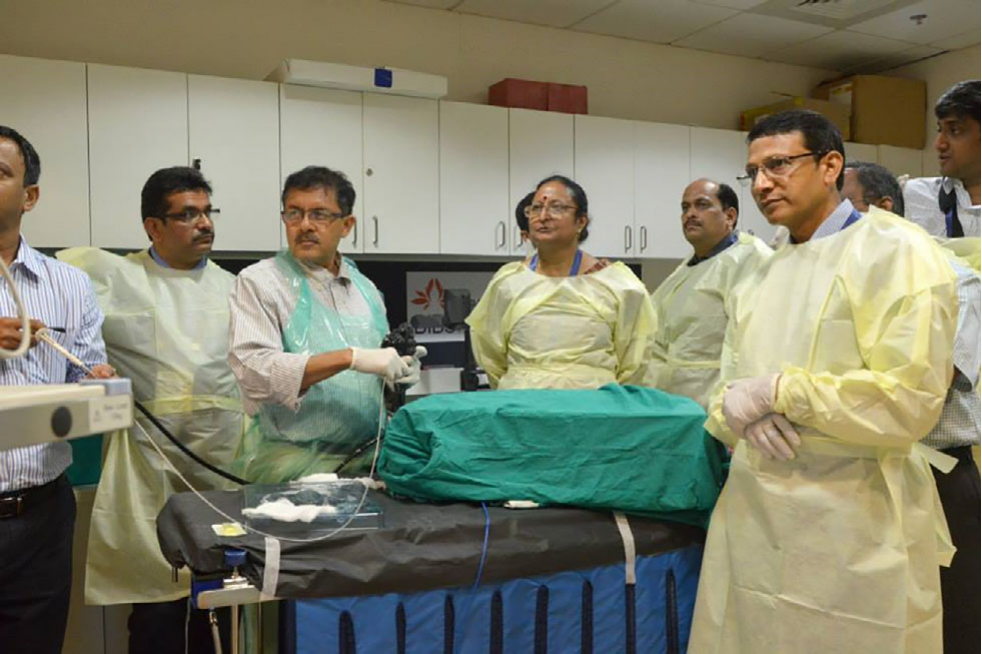
Animal model peroral endoscopic myotomy workshop at the Baldotta Institute of Digestive Sciences, Global hospital, Mumbai, India (2014).

In 2014, Dr Amit Maydeo, a leading international expert in therapeutic endoscopy from Mumbai, India decided to form the Asian POEM Consortium, for the purposes of conducting live workshops and training endoscopists to perform POEM. Representatives from several Southeast Asian countries were invited to join the Asian POEM Consortium, including Malaysia. This author (SM) was selected to represent Malaysia, due to prior experience in endoscopic submucosal dissection (ESD), which is a useful prerequisite for learning the POEM procedure (see later). The first Asian POEM Consortium workshop was conducted in the Baldotta Institute of Digestive Sciences, Global Hospital, Mumbai on 6 June 2014.^10^ The workshop consisted of international experts performing live POEM procedures on patients, and hands‐on training for POEM on ex‐vivo animal models specifically developed in India (Fig. 1).

**Figure 2:**
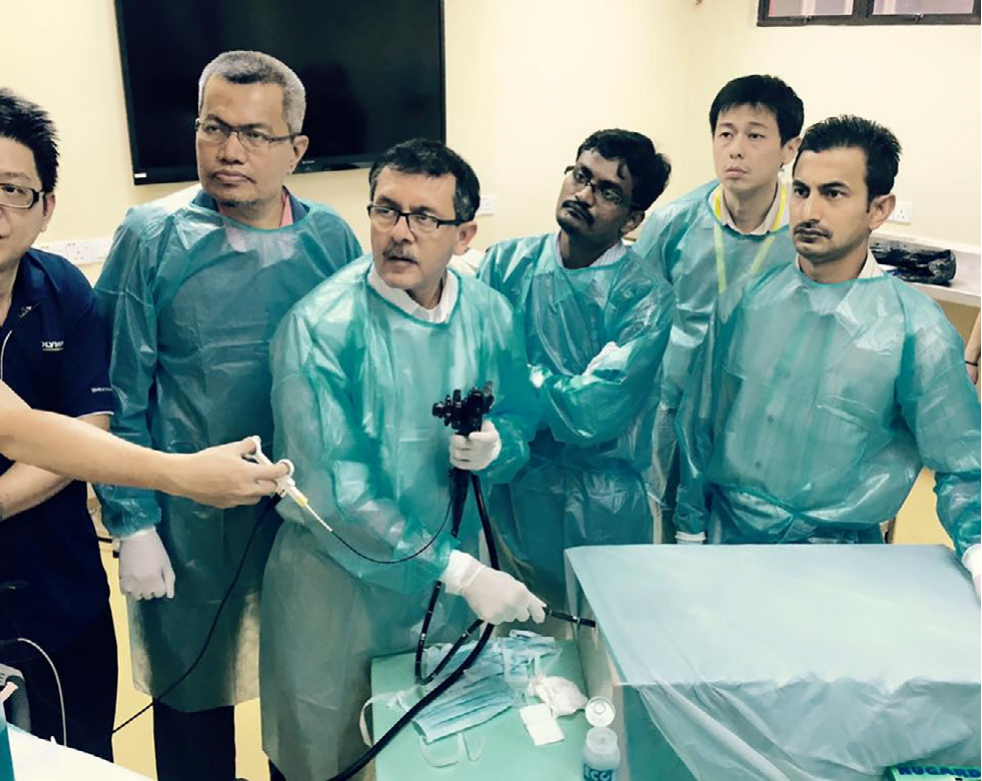
Animal model peroral endoscopic myotomy workshop at the University Malaya Medical Centre, Kuala Lumpur, Malaysia (2015).

Dr Maydeo was subsequently invited to the Live Endoscopy Workshop in Kuala Lumpur in April 2015, where he facilitated another POEM ex‐vivo animal model workshop for participants from Malaysia. At this workshop, several other endoscopists who were interested in the POEM procedure, particularly SHH and PCL, gained considerable experience with the POEM technique as well (Fig. 2). He performed POEM procedures for two patients with achalasia during the Live Workshop, the details of which were observed carefully by the authors, including specific considerations for general anesthesia by our in‐house anesthetist (MFH). This workshop in Kuala Lumpur was followed soon after by further training in ex‐vivo animal models (the second Asian POEM Consortium workshop in Mumbai, India and in Taiwan) in late 2015.^11^

With the skill set gained from the animal model workshops, coupled with background expertise in ESD and the surgical knowledge of an experienced Laparoscopic Upper GI surgeon, two Gastroenterologists (SM, SHH), and a Surgeon (PCL) came together to start a POEM service at the University Malaya Medical Centre. Our first, independent, POEM case was performed in November 2015, in a 45‐year‐old patient with Type II achalasia. The pre‐, peri‐, and post‐procedure care were conducted according to the protocol developed by Dr Maydeo.^12^ A posterior approach for submucosal tunneling and subsequent circular muscle myotomy was utilized, and dissection was performed using the Olympus Triangle Tip knife (Olympus, Tokyo, Japan). The total procedure duration was 3.5 h, and the patient developed some mild subcutaneous emphysema in the upper thorax, which settled spontaneously. A contrast swallow study 24 h after the procedure did not show any contrast leak at the site of hemostatic clips (mucosal incision) and the patient was discharged after commencing a soft diet 3 days later.

Following the success of our first POEM case, another five cases were attempted in 2016. However, we had difficulty with gaining access into the submucosal tunnel using a normal gastroscope transparent hood (Olympus, Tokyo, Japan) in several cases, resulting in unsuccessful procedures. In 2017, Prof Haruhiro Inoue, the world expert who had popularized POEM in humans, was invited as faculty for the University Malaya Medical Centre 2017 Live Endoscopy course. During this event, Prof Inoue conducted yet another ex‐vivo animal model POEM workshop with us, and we studied his technique of performing the POEM procedure in a patient during the live course. He introduced us to the tapered transparent hood (ST Hood, Fujifilm Corporation, Tokyo, Japan), which made it easier to gain access to the submucosal tunnel.^13^ Following this tip by the world's leading expert in POEM, we began using the new transparent hood for submucosal tunneling and this improved our success rate tremendously.

### Clinical outcomes of POEM in Malaysia

**Figure 3:**
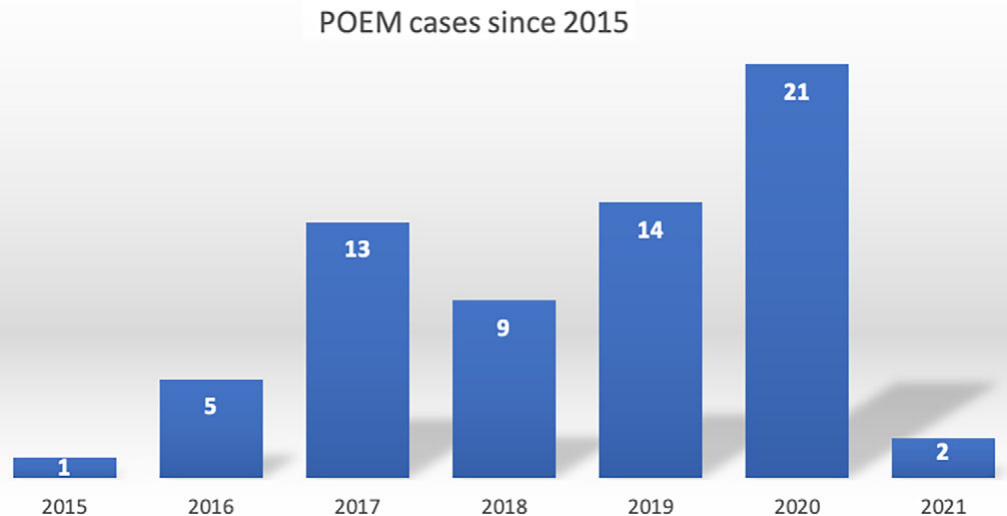
Number of peroral endoscopic myotomy cases per year, performed at University Malaya Medical Centre from 2015 to 2021.

To date, our center has been the only public institution to provide a POEM service in Malaysia. Since 2015, a total of 68 POEM cases for the indication of achalasia have been performed, but complete follow‐up data, over a mean of 19.3 ± 13.5 months, were available for 65 patients. The mean age of the patients was 41.7 ± 15.1 years and 55.4% were female. Table 1 highlights further clinical details of the patients. Figure 3 illustrates the gradual increase in POEM cases over the years, with an expected decrease in 2021 due to the COVID‐19 pandemic.

**Table 1:** Demographics and clinical details of peroral endoscopic myotomy (POEM) cases (*n* = 65)

Mean age, years (range)	41.7 ± 15.1 (16–76)
Gender	
Male	29 (44.6%)
Female	36 (55.4%)
Chicago classification	
Type 1	26 (40.0%)
Type 2	33 (50.7%)
Type 3	2 (3.1%)
Not available	4 (6.2%)
Sigmoid achalasia	
Type 1	3 (4.6%)
Type 2	0
Mean duration of procedure time, minutes(range)	125.8 ± 32.5 (60–180)
Mean length of myotomy, cm(range)	9.6 ± 2.1 (4–15)
Mean duration of hospital stay, day (range)	4.4 ± 1.4 (3–9)
Complications	14 (21.5%)
Minor	
Aspiration pneumonia	1 (1.5%)
Surgical emphysema or pneumomediastinum	5 (7.7%)
Intra‐procedural bleeding	4 (6.2%)
Mucosa injury	6 (9.2%)
Retention of foreign body	1 (1.5%)
Major	0
Eckardt score	
Mean Pre‐POEM, score	7.9 ± 2.5
Mean Post‐POEM (at 2 months), score	1.1 ± 1.9

The overall technical success of POEM, defined as a completion of all steps in the POEM technique, was 94.1%. Technical success for the first 20 cases was 85% and for the last 48 cases was 97.8%. The lower success rate in the initial 20 cases was largely due to the difficulties associated with gaining access to the submucosal tunnel, as highlighted previously. Among the last 48 cases, the was a single case of technical failure due to transient cardiopulmonary compromise secondary to air leak syndrome (pneumomediastinum) following myotomy. Due to inability to sustain adequate ventilation, further myotomy was aborted. The patient recovered uneventfully following mucosal closure. A pre‐ and post‐Eckardt score were measured among all cases for the duration of follow up.

The complication rate for POEM was 21.5%, all of which were minor and no patients required surgical intervention. Mucosal injury during submucosal tunneling occurred in six (9.4%) cases, and all cases were successfully treated with endoscopic hemostatic clips from within the lumen of the esophagus.

Clinical success (defined as a post‐procedure Eckardt score <3 or a reduction of >3 at 2 months) of POEM was observed among 57 of 61 (93.4%) patients who had a technically successful procedure. Post‐POEM gastroesophageal reflux was assessed by the requirement of proton pump inhibitor (PPI) at the time of follow up, data of which were available in 54 patients. Nine (16.7%) patients were requiring regular PPI, 11 (20.3%) were consuming on‐demand PPI, and 34 (63%) were not taking any PPI at the time of follow up.

## A multidisciplinary expertise for POEM

### Prior ESD experience

The essential skills required for a POEM procedure are the ability to initiate mucosal incision and tunnel entry, to dissect the submucosal tissue during tunnel creation, to secure bleeding using either electrosurgical knife or thermocoagulation device such as hemostatic grasper, to perform myotomy using electrosurgical knife, and to achieve closure of mucosal defect using hemostatic clips.^13^ To endoscopists who perform therapeutics within the lumen of the GI tract, these steps may be alien. However, for the endoscopist trained in ESD, performing therapeutics in the submucosal layer (also known as the third space), using hemostatic devices, electrosurgical knives, and hemostatic clips application, are “par for the course.”

ESD is generally regarded to be more challenging than POEM as it requires greater skill in scope handling and maneuvering.^14^ The skill gained in ESD from preventing thermal injury to the muscular layer by the electrosurgical knife is useful in preventing thermal injury to the esophageal mucosal layer during POEM. ESD endoscopists frequently have to deal with bleeding from large vessels arising from the submucosal layer during submucosal dissection. The only additional skill set for POEM that an ESD endoscopist may not be familiar with is that of performing a myotomy, which is fortunately not too difficult with electrosurgical dissection.

In a recent editorial on POEM learning curve studies, Modayil et al. reported that most operators at centers providing a POEM service had prior ESD or Heller's myotomy experience.^15^ As mentioned earlier, our center had been performing ESD for several years prior to initiating our POEM service. The results of our ESD experience had been reported previously,^16^ indicating a comparable outcome with large volume centers globally.

### Surgical knowledge and experience

The anatomy of the esophagus and mediastinum are well known to the Upper GI surgeon, as this area is regularly ventured into during laparoscopic or thoracoscopic surgery. Furthermore, they are able to appreciate the anatomy of the muscular layers of the esophagus better than the average gastroenterologist, who rarely venture beyond the submucosal layer from the lumen. To date, Malaysian Upper GI surgeons have had a reasonable experience treating achalasia with laparoscopic Heller myotomy. In a recent publication of laparoscopic Heller myotomy and anterior Dor fundoplication in 55 patients with achalasia in Malaysia, the authors reported a 12.7% minor complication rate, 67.3% complete resolution of dysphagia symptoms, and a high satisfaction rate among patients.^17^ The procedure involves splitting the longitudinal muscle fibers followed by the circular muscle fibers of the esophagus. The end point of the Heller's myotomy is to see the esophageal mucosa bulges out into the mediastinum after splitting both set of muscles. This is the exact reverse order of the steps in a POEM procedure.

The incidence of gastric cancer is low in Malaysia and Southeast Asia.^18^ Hence, the uptake and interest in ESD has been limited, apart from a few specialized centers.^16^ In contrast, achalasia is not that uncommon^1^ and hence relying solely on prior experience in ESD in this region will reduce the uptake of POEM in this region too. By combining the skill set of therapeutic endoscopists and surgical knowledge of an experienced laparoscopic and endoscopic Upper GI surgeon, we have shown that a POEM service can be successfully developed.

During the initial learning period, having an experienced surgeon in the team also helps alleviate the fear of the gastroenterologist if they encounter unfamiliar mediastinal anatomy or complications. The reassurance that any potential major complication can be dealt with immediately with a surgeon on the POEM team cannot be understated. This is particularly important during the initial phase of the learning curve to ensure that the procedure is not abandoned prematurely due to technical glitches or minor complications.

### Anesthetic support

In our institution, general anesthesia (GA) has been provided for POEM procedures in the endoscopic suite, rather than in an operating theater. Providing GA in a remote place has to be properly planned and carried out efficiently to avoid any mishaps. All patients receive intravenous (IV) amoxicillin clavulanate prior to induction of anesthesia, as per the protocol established by Dr Maydeo.^12^ With the patient in a supine position, standard monitoring and GA are administered. End‐tidal carbon dioxide (EtCO_2_) level is observed to increase in all patients (due to CO_2_ insufflation by the endoscope) and minute ventilation has to be optimized during the procedure to ensure near normal EtCO_2_ level. Common analgesic agents (opioid and non‐opioid) are administered peri‐operatively, while IV paracetamol and parecoxib are continued postoperatively until they can resume enteral analgesia. Postoperative nausea and vomiting are prevented by the administration of dexamethasone, ondansetron, and metoclopramide. The usage of deep neuromuscular blockade is crucial in POEM as it ensures an optimal endoscopic surgical condition. Sugammadex is administered to reverse the muscle relaxant and facilitate resolution of the insufflated CO_2_ effect. Advanced hemodynamic monitoring and continuous bladder drainage are rarely needed as the procedure duration is usually less than 2 h.

Aspiration pneumonia and CO_2_‐related complications are two main complications that concern anesthetists for a POEM procedure. Solid food residue in the esophagus and food debris around the vocal cords may predispose achalasia patients to aspiration pneumonia following GA for a POEM procedure. Preventive measures include modified rapid sequence induction anesthesia with rocuronium,^19^ a prolonged fasting period, and a diagnostic endoscopy to clear up the esophagus prior to GA. Pneumoperitoneum, subcutaneous emphysema, pneumothorax, and/or pneumomediastinum are recognized sequelae of CO_2_‐related complications.^7^, ^20^ Vigilant observation of the peak airway pressure and concurrent discussion with the endoscopist during POEM are important measures to detect and manage these complications. Although some of our patients did develop these CO_2_‐related complications (see above), all of them resolved conservatively. Overall, GA for POEM is safe and has a short learning curve for the experienced anesthetist. Vigilant monitoring and anticipation of complications will ensure a safe and successful POEM procedure.

## Conclusion

There is increasing evidence to support the role of POEM as the main therapeutic modality in the treatment of achalasia. Although it has gained popularity in many parts of the world, data from the Southeast Asian region appear to be lacking. In the absence of prior local expertise, we have highlighted that a POEM service can be developed through networking with regional experts, having necessary skill sets useful for the POEM procedure, attending animal model workshops, and working together in a multidisciplinary team. The POEM outcomes of a 21.5% minor complication rate and 93.4% clinical success rate are comparable to the published literature.^7^ We believe that our model may be useful for other Endoscopy Units in the region, which are performing advanced therapeutic endoscopy, to develop a POEM service too.

## Acknowledgement

The authors would like to thank Mrs Manjit Singh (Talvant Kaur) for her invaluable assistance in record keeping.
